# The effect of erythropoietin on neonatal hypoxic-ischemic encephalopathy: An updated meta-analysis of randomized control trials

**DOI:** 10.3389/fped.2022.1074287

**Published:** 2023-01-09

**Authors:** Jing-Jing Pan, Yue Wu, Yun Liu, Rui Cheng, Xiao-Qing Chen, Yang Yang

**Affiliations:** ^1^ First Affiliated Hospital, Nanjing Medical University, Nanjing, China; ^2^ Children's Hospital of Nanjing Medical University, Nanjing Children's Hospital, Nanjing, China

**Keywords:** erythropoietin, hypoxic-ischemic encephalopathy, meta-analysis, neonate, mild hypothermia

## Abstract

**Objective:**

Erythropoietin (EPO) seems to have a good application prospect both in experimental models and patients with hypoxic ischaemic encephalopathy (HIE). Data regarding the effect of EPO on death or neurodevelopmental impairment are conflicting.

**Methods:**

A search was conducted by two investigators involved in this research in PubMed, Embase, and Cochrane databases for studies in English, in Wanfang, VIP, and Cnki databases for Chinese studies (all last launched on 2022/08/31). Ultimately, we identified 11 original studies, including the EPO group (*n* = 636) and the control group (*n* = 626). Odds ratio (OR) and weighted mean difference were calculated using a random effects or fixed effects model, depending on the data type and heterogeneity of the included studies.

**Results:**

1. The comparison of effectiveness of EPO treatment on HIE: (1) With respect to death, data showed no significant difference between EPO and control groups (OR = 0.97, 95% CI, 0.66–1.43; *P* = 0.88); Considering the additional effect of mild hypothermia treatment (MHT), no significant difference was found between EPO + MHT/control + MHT groups either (OR = 1.09, 95% CI, 0.69–1.73; *P* = 0.72); With respect to the interference of different routes of medication administration, Meta-analysis further showed no difference between intravenous EPO/control groups (OR = 1.13, 95% CI, 0.70–1.82; *P* = 0.62). (2) With respect to cerebral palsy, the analysis showed no significant difference (OR = 0.76, 95% CI, 0.50–1.15; *P* = 0.20); Considering the effect of MHT and routes of medication administration, data further showed no difference between EPO group and control group (OR = 1.26, 95% CI, 0.73–2.19; *P* = 0.41). (3) Regarding epilepsy, no significant difference was found (OR = 0.49, 95% CI, 0.20–1.19; *P* = 0.12). MR abnormality was less common in EPO group (OR = 0.39, 95% CI, 0.19–0.79; *P* = 0.008). 2. The comparison of possible adverse events of EPO: EPO treatment would not increase the risk of thrombocytopenia, hypotension, and hepatic and kidney injury.

**Conclusions:**

This meta-analysis showed that EPO treatment is not beneficial for reducing death and improving neurological impairment, though it would not increase the risk of adverse events.

## Introduction

Perinatal asphyxia is one of the important causes of death at any age over the world (1). Severe asphyxia could cause hypoxic ischaemic encephalopathy (HIE). And the incidence of HIE in low-income and middle-income countries is 10–20 times higher than that in high-income countries (2, 3). Outcomes of HIE vary from recovery to death or survival with neurological disability (4, 5). In China, though there haven't been detailed data from large sample and multi-center study, the incidence of HIE in live births reported by different single centers has reached from 0.69% to 0.95% (6, 7). Therapy for this disease mainly depends on effective support, cerebral protection and early mild hypothermia treatment (MHT). However, even with MHT, neurologic impairment or death is still common, occurring in at most 40% of newborns in developing countries (8, 9). So, the development of new treatments for HIE has been urgently needed.

Recombinant human erythropoietin (EPO), a cytokine known as its role in erythropoiesis, is a promising neuroprotective treatment in brain injury. In animal models of neonatal hypoxic-ischemic brain injury, EPO could alleviate impairment and improve sensorimotor function (10, 11). In consistent with animal studies, two small randomized controlled studies (RCTs) found EPO improved short-term neurological outcomes in HIE neonates without hypothermia therapy (12, 13). A placebo-controlled, double-blind RCT study in HIE neonates with MHT demonstrated EPO-treated patients had minor brain injury through MRI scan and improved motor outcomes at 12 months old (14). A meta-analysis at 2019 also showed EPO administration in neonates with perinatal HIE reduces the risk of brain injury, cerebral palsy and cognitive impairment (15). However, a new larger multicenter RCT performed by Wu et al. found the administration of erythropoietin to newborns undergoing therapeutic hypothermia for HIE did not result in a lower risk of death or neurodevelopmental impairment (16).

So, in view of the contradiction and uncertainty, an updated meta-analysis including the latest literature is performed to evaluate the potential effect of EPO on neonatal HIE.

## Methods

### Study selection

Guidelines from the preferred reporting items for systematic reviews and meta-analysis (PRISMA) were followed for this study (17). In order to screen eligible studies published since each database was established, a search was conducted by two investigators in PubMed, Embase, and Cochrane databases for studies in English, in Wanfang, VIP, and Cnki databases for Chinese studies (databases were last launched on 2022/08/31). The following search terms were employed: “hypoxic-ischemic encephalopathy,” or “HIE,” and “EPO”, or “erythropoietin.”.

The inclusion criteria of this meta-analysis were as follows: (1) RCT involving HIE with EPO treatment; (2) The results reported on the effectiveness of EPO treatment on HIE; (3) Human clinical studies. Exclusion criteria: (1) Different study design: non-RCT studies, case reports, reviews, meta-analysis, protocol, case-control studies, animal experiments; (2) Not available for enough outcome information; (3) Studies without good design. Any discrepancies were independently resolved by a third investigator involved in this research. This meta-analysis was registered in PROSPERO (CRD42022356809).

### Data abstraction

The quality of all included studies was assessed by the consolidated standards of reporting trials (CONSORT) items and Jadad score (18–20). Finally, from the full-text and corresponding supplement information, the following eligibility items were collected and shown in tables for each study: author, year of publication, study time, participants, gestation age, birthweight, EPO administration method (dose, frequency and course), hypothermia therapy, HIE severity, inclusion, exclusion, primary outcomes, randomization, blinding, Jadad score, and CONSORT items.

Subsequently, the outcomes were divided into two parts. First was the comparison of effectiveness of EPO treatment on HIE (including death, cerebral palsy, epilepsy and MR abnormality). Second, with respect to the possible adverse events of EPO, blood cell count change, hepatic injury, kidney injury and hypotension were compared between EPO and control groups. The longest follow-up of the included study was three years (16).

### Statistical analysis

For each outcome, either odds ratio (OR) or weighted mean difference (WMD) with the 95% confidence interval (95% CI) was calculated, depending on the data type. Both a fixed effects model and a random effects model were considered. For each meta-analysis, the *χ*^2^-based *Q* statistic test (Cochran *Q* statistic) (21) was applied to test for heterogeneity, and the *I*^2^ statistic was also used to quantify the proportion of the total variation attributable to heterogeneity (22). For *P* values < 0.05 or *I*^2^ > 50, the assumption of homogeneity was assumed to be invalid, and the random-effects model was used; for *P* value ≥ 0.05 and *I*^2 ^≤ 50, data were assessed using the fixed-effects model. Publication bias was investigated by funnel plot, and an asymmetric plot suggested possible publication bias. Statistical analyses were performed using Review Manager 5.2 (Cochrane Collaboration, Nordic Cochrane Centre). A two-tailed *P* value of less than 0.05 was deemed statistically significant.

## Results

### Demographic characteristics of the studies

**Figure 1:**
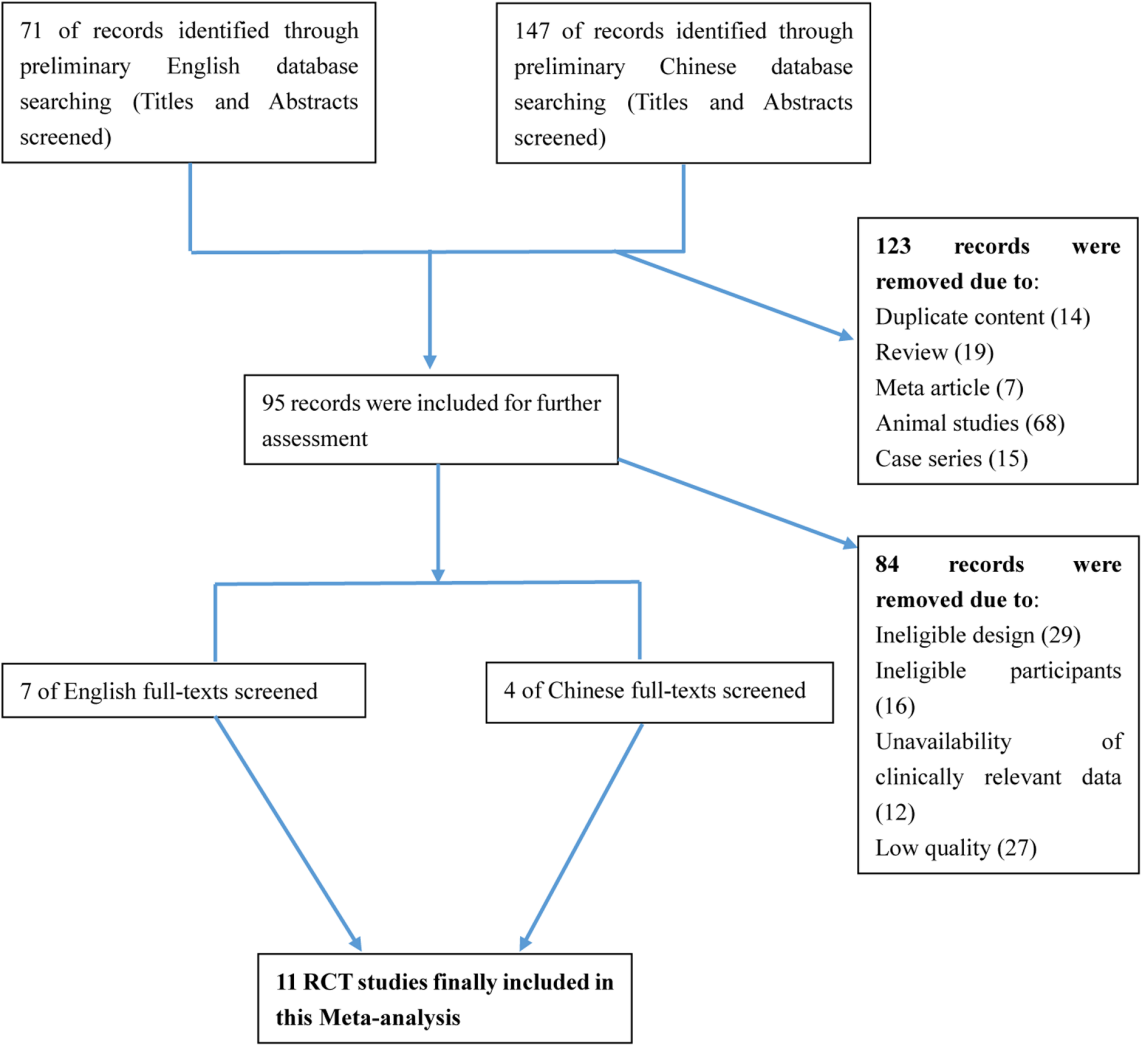
Flow diagram of selection of studies for inclusion in the meta-analysis.

**Figure 2:**
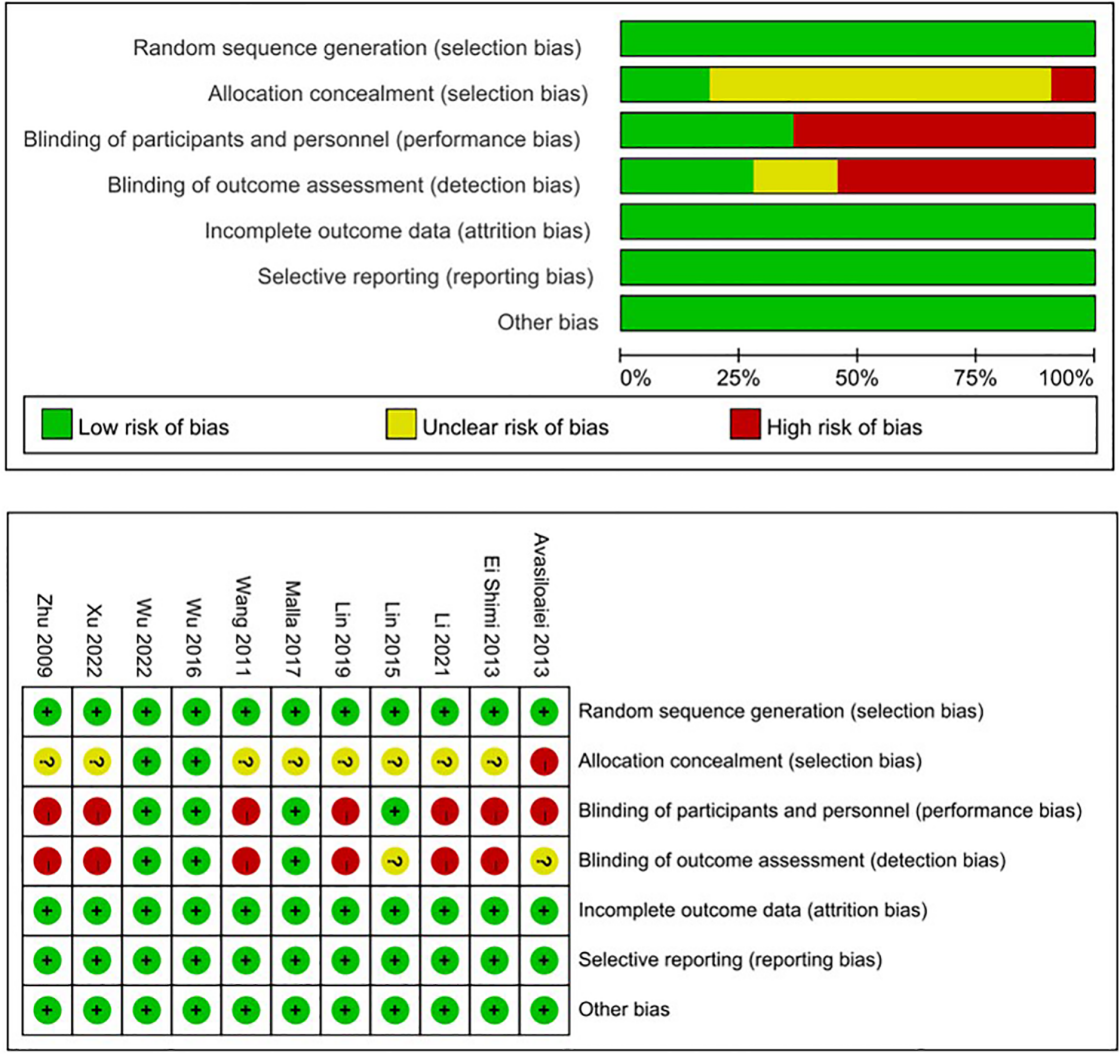
Risk of bias by revMan software.

After searching the above databases, 218 potentially relevant studies were obtained. Details of the searching process are shown in Figure 1. A search of other aforementioned databases did not identify any additional eligible studies. Ultimately, we identified 11 original RCT studies (7 in English and 4 in Chinese) (12, 14, 16, 23–30), including the EPO group (*n* = 636) and the control group (*n* = 626) (Table 1). The quality of all studies included into this meta-analysis was assessed by Jadad score, CONSORT items and Risk of bias provided by RevMan software (Table 2 and Figure 2).

**Table 1:** Demographic characteristics of trials included in the meta-analysis.

Study	Country/Study time	EPO/Control (*n*)	Gestational age (weeks)	Birthweight (*g*)	EPO administration	Age at EPO therapy (*d*)	Course (frequency/days)	Hypothermia therapy	HIE severity	Main inclusion criteria	Main exclusion criteria	Outcomes
Avasiloaiei, 2013 (23)	Romania (2010.01–2011.09)	22/23	≥37	(Mean) 3278	1,000 U/kg*d (IH)	1	3/3	N	NR	Perinatal asphyxia (AAP criteria)	Severe malformation; Rh incompatibility	Mortality; Neurodevelopmental assessment; SOD; GPx; TAS
El Shimi, 2013 (24)	Egypt (2007.09–2010.02)	10/10	>36	>1800	1,500 U/kg*d (IH)	1	1/1	Yes	Moderate to severe	pH ≦ 7.0 or BE ≦ −16 mmol/l	Severe malformation; Infection; Severe growth restriction; Metabolic disease	Mortality; NSE; BDNF; MRI score
Malla, 2017 (25)	India (2012.12–2015.11)	50/50	≥37	EPO2902 ± 444/Control3136 ± 660	500 U/kg*d (IV)	1	5/9	No	Moderate to severe	pH < 7.0 or BE≦−16 mmol/l or 10 min Apgar <5 or Ventilation ≥10 min	Severe malformation; Infection; Severe growth restriction; Metabolic disease	Mortality; Disability; Neurodevelopmental assessment; Organ function damage
Zhu, 2009 (12)	China (2003.08–2007.01)	76/82	>37	>2,500	300–500 U/kg*d (IV)	1–2	8/14	No	Moderate to severe	5 min Apgar ≦5 or need resuscitation at 10 min	Severe malformation; Intra cranial hemorrhage; Postnatal age of >48 h; Hypothermia	Mortality; Disability; Neurodevelopmental assessment; Whole blood count
Li, 2021 (26)	China (2017.03–2019.07)	46/46	≥36	EPO3020.29 ± 430.71/Control3158.28 ± 421.47	1,000 U/kg*d (IV)	1	8/14	Yes	Moderate to severe	pH ≦ 7.1 or BE≦−16 mmol/l or 5 min Apgar <5	Severe malformation; Malformation; Severe infection; Intra cranial hemorrhage; Severe anemia	Mortality; Neurodevelopmental assessment; Organ function damage
Wang, 2011 (27)	China (2009.04–2010.08)	35/35	≥37	EPO32401 ± 160/Control3215 ± 1215	200 U/kg*d (IV)	1	6–12/14–28	No	Moderate to severe	pH < 7.0 or 5 min Apgar <5	Severe malformation; Intra cranial hemorrhage	Neurodevelopmental assessment
Lin, 2015 (28)	China (2012.02–2015.03)	25/19	≥36	EPO3357 ± 349/Control3256 ± 398	1,000 U/kg*d (IV)	1	7/14	Yes	Moderate to severe	pH ≦ 7.1 or BE≦−16 mmol/l or 5 min Apgar <5	Severe malformation; Intra cranial hemorrhage; Severe infection; Severe anemia	Organ function damage; Whole blood count
Lin, 2019 (29)	China (2013.03–2016.07)	49/49	≥37	EPO3400 ± 300/Control3500 ± 300	300 U/kg*d (IH)	1	36/84	No	NR	pH ≦ 7.1 or BE ≦ −16 mmol/l or 5 min Apgar <5	Severe malformation; Intra cranial hemorrhage; Organ failure	SOD; MDA; Neurodevelopmental assessment
Wu, 2016 (14)	United States (2012.01–2012.11)	24/26	≥36	EPO3556 ± 618/Control3243 ± 512	1,000 U/kg*d (IV)	1	5/7	Yes	Moderate to severe	pH < 7.0 or BE ≦ −15 mmol/l or 10 min Apgar <5	Severe malformation; Severe growth restriction; Moribund condition	Neurodevelopmental assessment; Organ function damage; MRI score
Wu, 2022 (16)	United States (2017.01–2019.10)	257/243	≥36	EPO3332 ± 572/Control3414 ± 614	1,000 U/kg*d (IV)	1	5/7	Yes	Moderate to severe	pH < 7.0 or BE ≦ −15 mmol/l or 10 min Apgar <5	Severe malformation; Severe growth restriction; Hematocrit >65%	Mortality; Neurodevelopmental assessment; Organ function damage
Xu, 2022 (30)	China (2020.05–2021.05)	45/45	NR	NR	1,000 U/kg*h (IM)	NR	7/1	Yes	Mild to severe	NR	NR	Mortality; SOD; GPx; AOPP; ROS

IH, subcutaneous injection; IV, intravenous injection; IM, intramuscular injection; NR, not reported; AAP, american academy of pediatrics; SOD, superoxide dismutase; GPx, glutathione peroxidase; TAS, total antioxidant status; NSE, neuron-specific enolase; BDNF, brain-derived neurotrophic factor; MDA, malondialdehyde; AOPP, advanced oxidation protein products; ROS, reactive oxygen species.

**Table 2:** Report quality of trials included in the meta-analysis.

Study	Title and Abstract	Participant Flow	Baseline Data	Randomization	Blinding	Follow-up	CONSORT Items (22)	Jadad Score (5)
Avasiloaiei, 2013 (23)	Yes	Yes	Yes	Yes	No	Yes	16	3
El Shimi, 2013 (24)	Yes	No	No	Yes	No	Yes	16	3
Malla, 2017 (25)	Yes	Yes	Yes	Yes	Yes	Yes	19	5
Zhu, 2009 (12)	Yes	Yes	Yes	Yes	No	Yes	19	4
Li, 2021 (26)	Yes	No	Yes	Yes	No	Yes	16	3
Wang, 2011 (27)	Yes	No	Yes	Yes	No	Yes	17	4
Lin, 2015 (28)	Yes	No	Yes	Yes	Yes	No	20	5
Lin, 2019 (29)	Yes	No	Yes	Yes	No	Yes	16	3
Wu, 2016 (14)	Yes	Yes	Yes	Yes	Yes	Yes	20	4
Wu, 2022 (21)	Yes	Yes	Yes	Yes	Yes	Yes	21	5
Xu, 2022 (30)	Yes	Yes	No	Yes	No	No	16	3

### The comparison of effectiveness of EPO treatment on HIE (including death, cerebral palsy, epilepsy and Mr abnormality)

**Figure 3:**
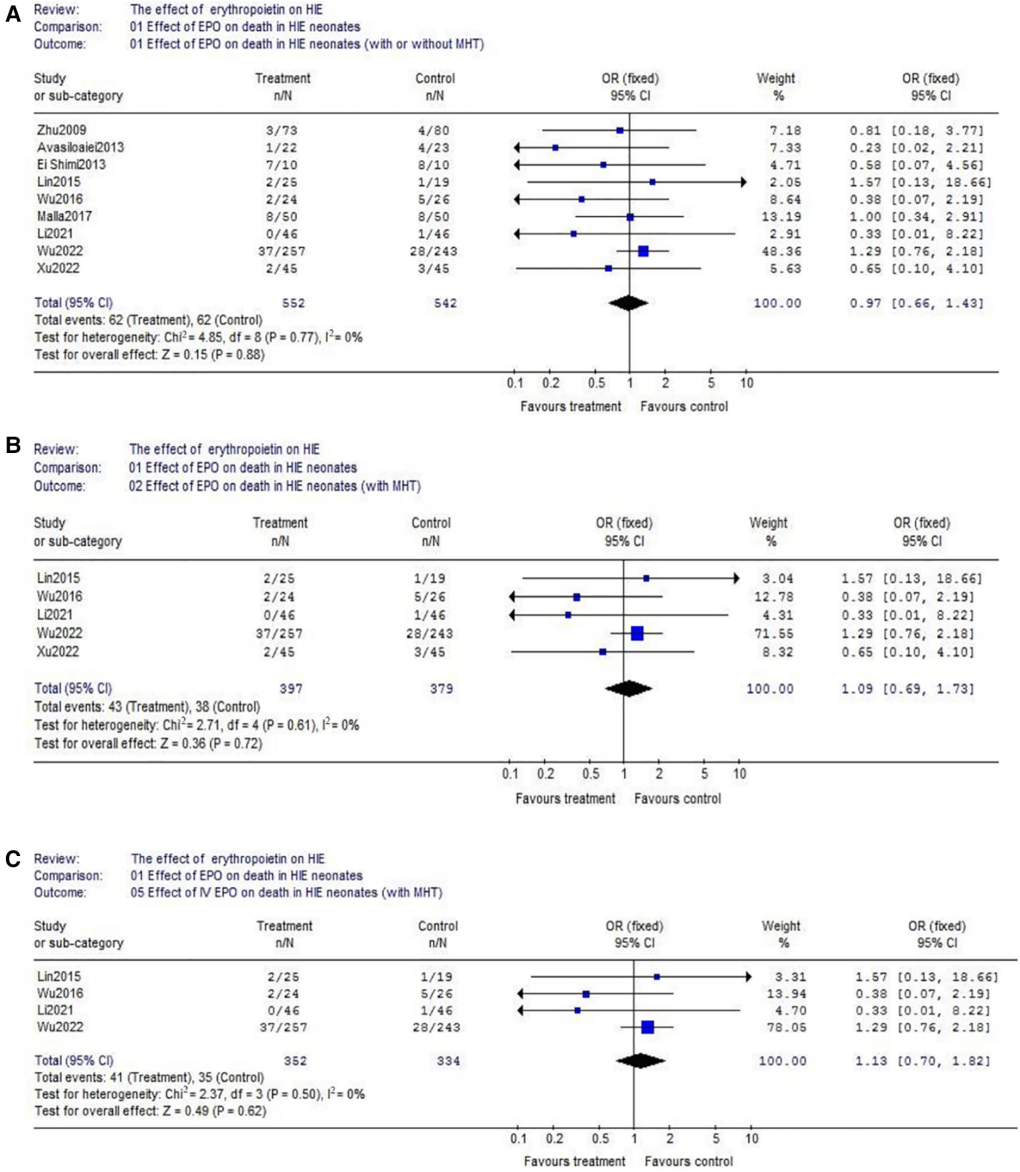
The comparison of effectiveness of EPO treatment on death. (**A**) Effect of EPO on detah in HIE neonates (with or without MHT); (**B**) Effect of EPO on detah in HIE neonates (with MHT); (**C**) Effect of IV EPO on detah in HIE neonates (with MHT).

**Figure 4:**
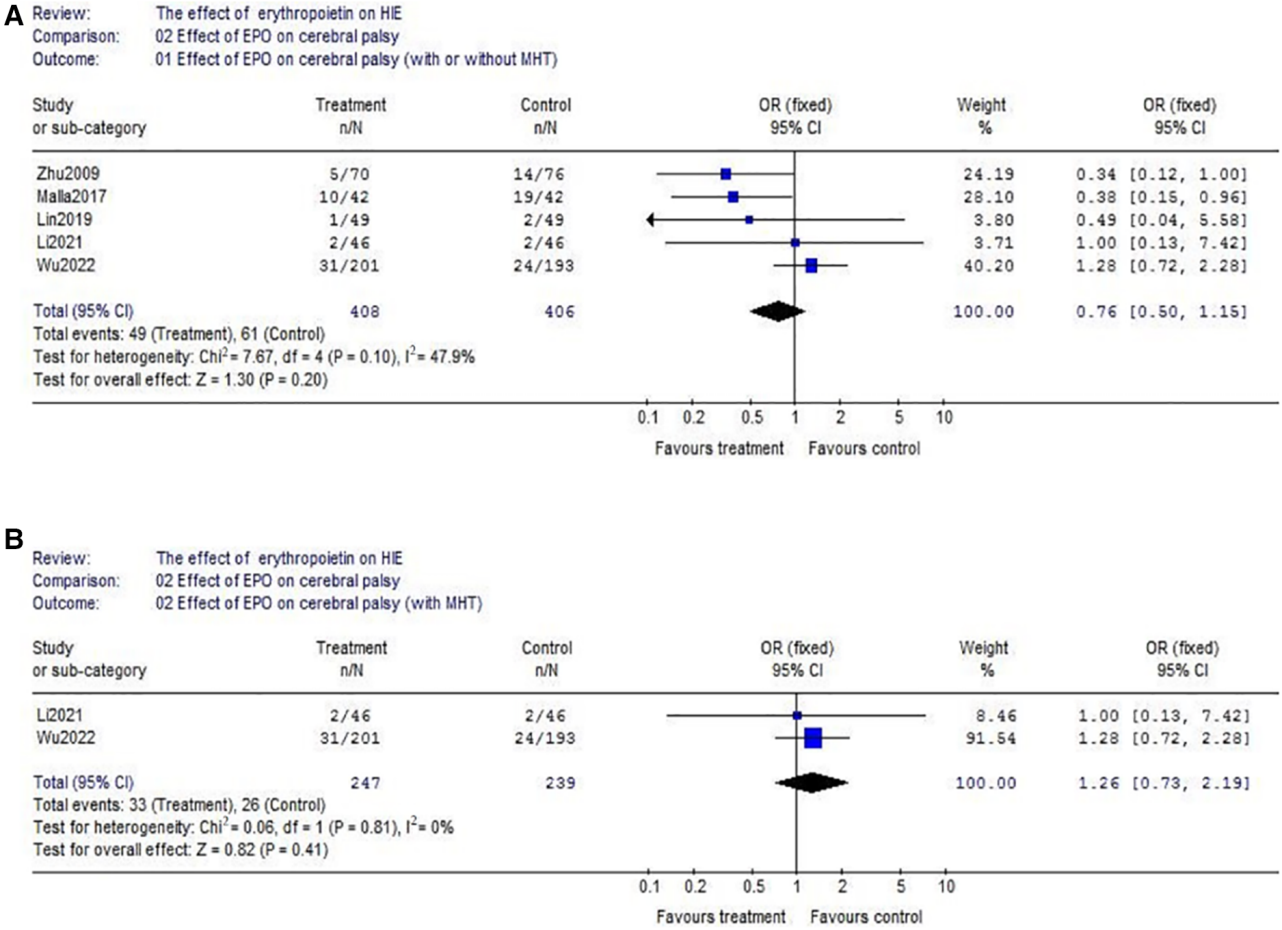
The comparison of effectiveness of EPO treatment on cerebral palsy. (**A**) Effect of EPO on cerebral palsy (with or without MHT); (**B**) Effect of EPO on cerebral palsy (with MHT).

**Figure 5:**
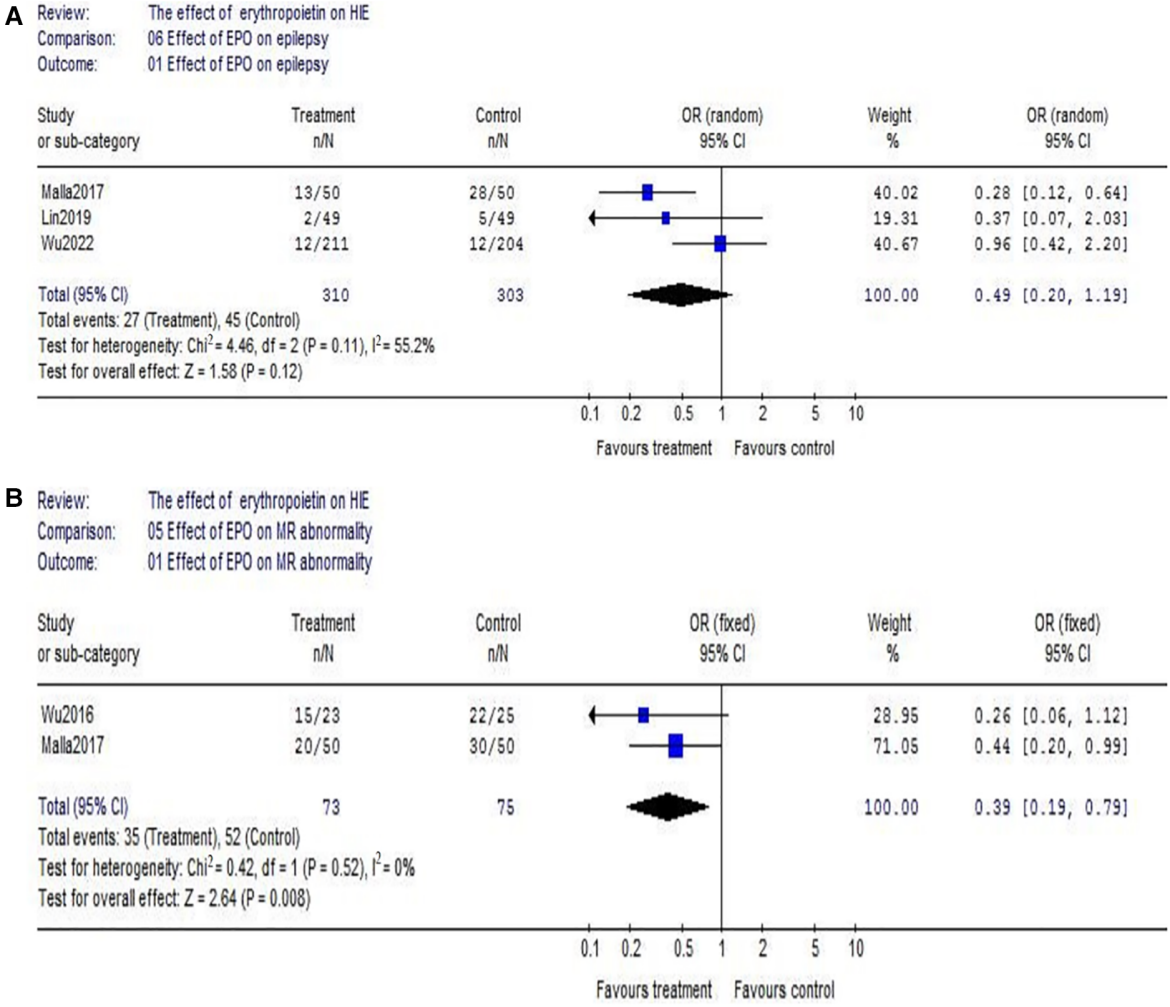
The comparison of effectiveness of EPO treatment on epilepsy and MR abnormality. (**A**) Effect of EPO on epilepsy; (**B**) Effect of EPO on MR abnormality.

(1) With respect to death, data were reported by 9 trials (EPO group/control group = 552/542) (Figure 3A). There wasn't heterogeneity (*χ*^2^ = 4.85, *P* = 0.77; *I*^2^ = 0%). Data showed no significant difference (OR = 0.97, 95% CI, 0.66–1.43; *P* = 0.88); Considering the additional effect of MHT, we subsequently analyzed EPO treatment with hypothermia. Data also showed no significant difference between EPO/control groups (OR = 1.09, 95% CI, 0.69–1.73; *P* = 0.72) (Figure 3B); With respect to the interference of different routes of medication administration, patients treated with intravenous (IV) EPO combined with MHT were further analyzed (IV EPO group/control group = 352/334) (Figure 3C). There wasn't heterogeneity (*χ*^2^ = 2.37, *P* = 0.50; *I*^2^ = 0%). Meta-analysis showed no difference between IV EPO/control groups (OR = 1.13, 95% CI, 0.70–1.82; *P* = 0.62).
(2) With respect to cerebral palsy, 5 studies were included into this meta-analysis (EPO group/control group = 408/406). There was no significant heterogeneity among the trials (*χ*^2^ = 7.67, *P* = 0.10; *I*^2^ = 47.9%). The analysis showed that there was no significant difference (OR = 0.76, 95% CI, 0.50–1.15; *P* = 0.20) (Figure 4A); Considering the effect of MHT, we analyzed the effect of EPO combined with MHT on cerebral palsy which includes two RCTs. Data showed no difference between EPO group and control group (OR = 1.26, 95% CI, 0.73–2.19; *P* = 0.41) (Figure 4B); With respect to the interference of different routes of medication administration, studies with IV EPO combined with MHT were further analyzed. But the included studies (Li2021 and Wu2022) were the same as showed in Figure 4B. So, no further analysis was needed.
(3) Regarding epilepsy, there were 3 eligible studies included (EPO group/control group = 310/303), and significant heterogeneity was detected among these trials (*χ*^2^ = 4.46, *P* = 0.11; *I*^2^ = 55.2%). No significant difference was found in the comparison of the two groups (OR = 0.49, 95% CI, 0.20–1.19; *P* = 0.12) (Figure 5A). Regarding MR abnormality, there were 2 eligible studies included (EPO group/control group = 73/75), and there was no significant heterogeneity (*χ*^2^ = 0.42, *P* = 0.52; *I*^2^ = 0%). MR abnormality were less common in EPO group (OR = 0.39, 95% CI, 0.19–0.79; *P* = 0.008) (Figure 5B).

### The comparison of possible adverse events of EPO (blood cell count change, hepatic injury, kidney injury and hypotension)

**Figure 6:**
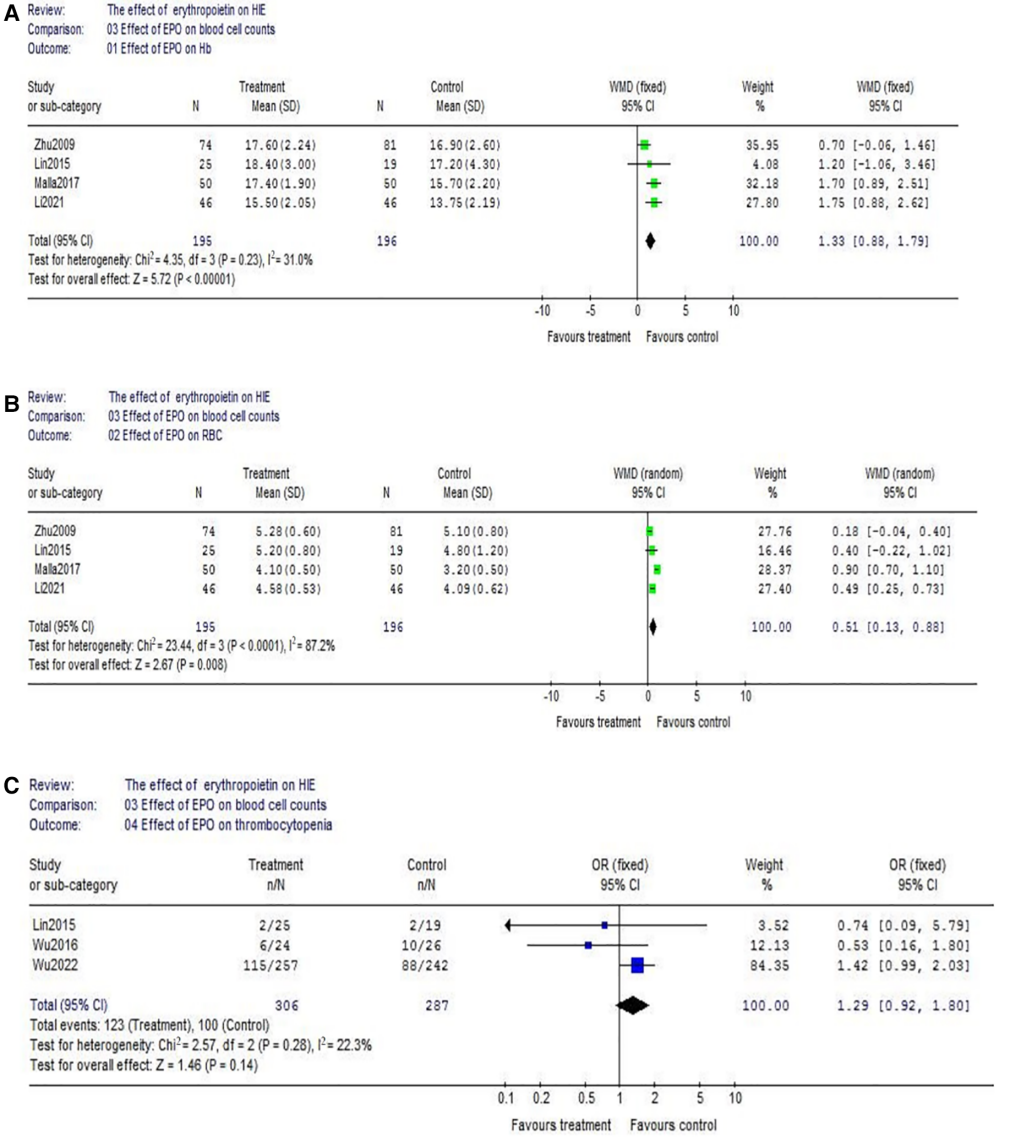
Effect of EPO on blood cell count change. (**A**) Effect of EPO on Hb; (**B**) Effect of EPO on RBC; (**C**) Effect of EPO on thrombocytopenia.

**Figure 7:**
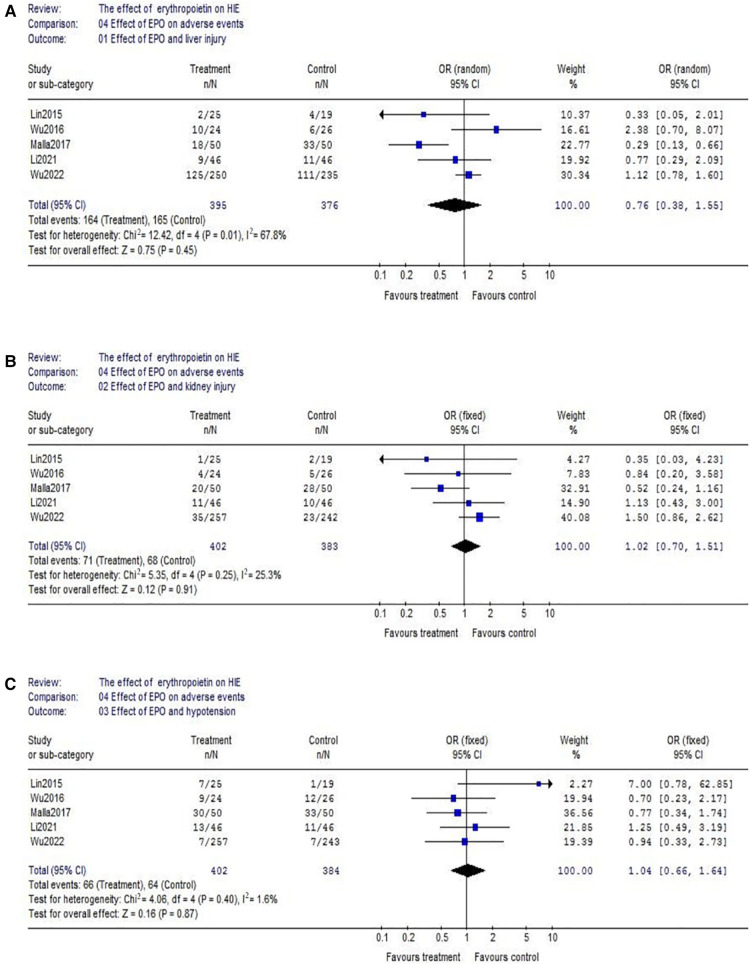
Effect of EPO on hepatic injury, kidney injury and hypotension. (**A**) Effect of EPO and liver injury; (**B**) Effect of EPO and kidney injury; (**C**) Effect of EPO and hypotension.

(1) Data of the comparison of hemoglobin (Hb) between EPO group and control group were reported by 4 studies (EPO group/control group = 195/196). There was no significant heterogeneity among these trials (*χ*^2^ = 4.35, *P* = 0.23; *I*^2^ = 31.0%). The result showed neonates with EPO therapy had higher Hb level (WMD = 1.33, 95% CI, 0.88–1.79; *P* < 0.00001) (Figure 6A); The similar result could be found in red blood cell (RBC) count. (EPO group vs. control group, WMD = 0.51, 95% CI, 0.13–0.88; *P* = 0.008) (Figure 6B); Considering the effect of EPO on platelet (PLT) count, 3 studies were included. Data showed no significant difference between EPO/control groups (OR = 1.29, 95% CI, 0.92–1.80; *P* = 0.14) (Figure 6C).
(2) Regarding hepatic injury, there were 5 eligible studies included (EPO group/control group = 395/376), and significant heterogeneity was detected among these trials (*χ*^2^ = 12.42, *P* = 0.01; *I*^2^ = 67.8%). The analysis showed that there was no significant difference (OR = 0.76, 95% CI, 0.38–1.55; *P* = 0.45) (Figure 7A); Considering the effect on kidney injury, we included five RCTs. Data showed no significant difference between EPO/control groups (OR = 1.02, 95% CI, 0.70–1.51; *P* = 0.91) (Figure 7B); Considering the effect on blood pressure, then we analyzed hypotension which includes 5 RCTs. Data also showed no significant difference between EPO group/control groups (OR = 1.04, 95% CI, 0.66–1.64; *P* = 0.87) (Figure 7C).

### Publication bias

**Figure 8:**
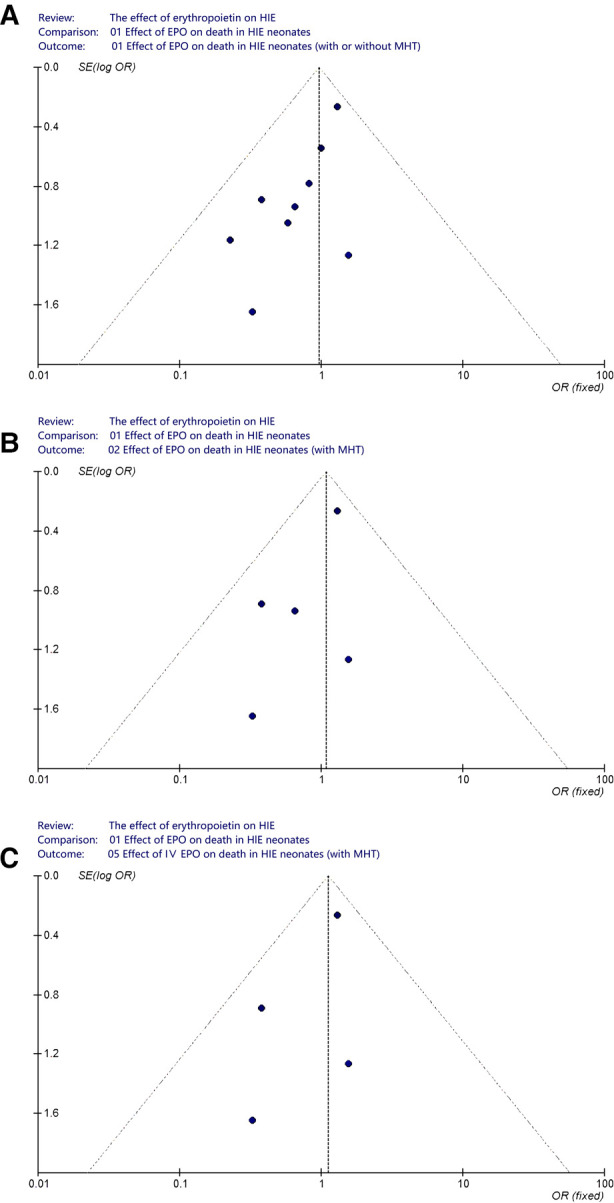
Funnel plot to assess publication bias. (**A**) Effect of EPO on death in HIE neonates (with or without MHT); (**B**) Effect of EPO on death in HIE neonates (with MHT); (**C**) Effect of IV EPO on death in HIE neonates (with MHT).

A funnel plot was performed in order to assess the potential publication bias in this meta-analysis. In analyzing the effect of EPO treatment on death (regardless of MHT and routes of medication administration), we visually evaluated the symmetry of funnel plot shape and found obvious evidence of asymmetry (Figure 8A). Considering the interference effect of MHT and administration routes, we further evaluated the symmetry of funnel plot shape in neonates with MHT and neonates with IV EPO, respectively. No obvious asymmetry was found anymore (Figures 8B,C).

## Discussion

EPO is first known for the haematological function. After an ischemic insult, the transcription of the Hypoxia Inducible Transcription Factor (HITF) induced by hypoxia evokes an increase level of EPO in kidney (31). EPO exhibits intracellular protective effects after the ischemia-reperfusion damage, such as decreasing oxidative stress, apoptosis and blood brain barrier injury (32). Furthermore, EPO could stimulate neurogenesis, angiogenesis and neuronal plasticity after the ischemic damage (33). In varying degrees, EPO has shown anti-apoptotic (34, 35), antioxidant (36, 37) and anti-inflammatory properties (38–40) both in animal models and neonates.

In many researches comparing EPO vs. control, EPO treatment showed a lower mortality and improved neurologic outcomes. For example, Avasiloaiei et al. found the motility was lower in EPO group compared with control group (5% vs. 17%) (23). And report from Elmahdy et al. showed the administration of EPO to infants with HIE was associated with significant decreases in endogenous nitric oxide, decreases in seizure activity, and improved neurodevelopmental outcomes to 6 months old (13). But, not a few studies further reported EPO did not helps reduce mortality and neurological impairment (12, 25). Considering the interference of MHT and routes of medication administration, in this meta-analysis we analyzed the effect of EPO on death in three conditions (Figures 3A–C). However, the beneficial effect of EPO treatment in preventing death was not proven through our analysis. As a matter of fact, in the recent large RCT performed by Wu et al. (16), they overturned the conclusion of their previous paper published in 2016 (14). In their new trial (EPO *n* = 257 vs. control *n* = 243), multiple high doses of EPO administered (1,000 U/kg*5d) during the first week of age to newborns undergoing MHT for HIE did not result in a lower risk of death or neurodevelopmental impairment.

As for neurodevelopmental impairment (including cerebral palsy and epilepsy), no significant beneficial effect of EPO was found in our study either. It is not completely consistent with previous reports. For example, the meta-analysis at 2019 showed EPO was helpful to decrease incidence of cerebral palsy (15). But, the above meta-analysis only included 2 small RCTs which limits its credibility. Moreover, it did not take into account the interference caused by different routes of administration. In fact, latest large-scale study has shown that EPO treatment does not have evident benefits (16). The National Institute of Child Health and Human Development Neonatal Research Network completed a randomized, controlled trial of early Epo and iron therapy in preterm infants ≦1,250 g. A total of 172 extremely low birthweight infants were enrolled (87 EPO and 85 placebo/control). There were no differences between groups with respect to the percentage of infants with Bayley-II Mental Developmental Index <70 (34% EPO and 36% placebo/control), moderate to severe cerebral palsy (16% EPO and 18% placebo/control) or the percentage of infants with any of the above-described neurodevelopmental impairments (42% EPO and 44% placebo/control) (41). Based on the above reasons, it seems not reliable that EPO could alleviate neurodevelopmental impairment. Though the occurrence of MR abnormality seems reduced in our meta-analysis (Figure 5B), the included sample size is relatively small (EPO group *n* = 73 vs. control group *n* = 75).

In addition to the above interference factors, evidence from animal models have shown that EPO should be administered at high doses within 6 h after the onset of brain injury to reach an enough neuroprotective effect (42). In contrast, the administration time of most studies was over 6 h or not described clearly. In addition, the dose and frequency were also different among RCTs. Concerning the time interval, previous animal model showed the area under the curve should achieve >140,000 mU*h/ml and C_max_ > 10,000 mU/ml after 48 h (43). Wu et al. further proved in the Phase II RCT, the dosing interval was shortened to 1,000 U/kg every 24 h for the first two days of therapy in order to ensure adequate exposures after injury (44). But, some studies still used lower dose (300–500 U/kg) or longer dosing interval (48 h) (Table 1). Besides, the optimal duration of EPO dosing after hypoxic-ischemic injury has not been known yet. Considering those confounding factors, more well designed large RCTs are urgently needed to eliminate the above interference.

EPO is traditionally used in preterm neonates for the treatment of anemia of prematurity. Binding of EPO to receptors on erythroid progenitor cells causes an increase in red blood cell mass. So, we also evaluated the effect on peripheral blood cell count. And it turns out EPO can significantly improve the level of red blood cells and hemoglobin. But, it did not cause thrombocytopenia and hypotension. In addition, EPO treatment did not lead to hepatic and kidney injury in HIE neonates.

In spite of the aforementioned concerns, we must note additional limitations to some included researches. For example, in analyzing the effect of EPO treatment on death (regardless of MHT and routes of medication administration), we visually evaluated the symmetry of funnel plot and found obvious asymmetry. It may be related to the fact that negative research results had not been published. In addition, methods of specific randomization and detailed blinding were not included in the published reports. Moreover, patients were followed up for different time courses. Besides, some studies adopted different developmental assessment scores, which makes it difficult to evaluate and analyze.

In conclusion, our meta-analysis showed that EPO treatment would not increase the risk of adverse events (thrombocytopenia, hypotension, and hepatic and kidney injury). But it is not beneficial for reducing death and improving neurological impairment according to the existing literature.

## Data Availability

The original contributions presented in the study are included in the article/Supplementary Material, further inquiries can be directed to the corresponding author.
